# Pea hull fiber supplementation does not modulate uremic metabolites in adults receiving hemodialysis: a randomized, double-blind, controlled trial

**DOI:** 10.3389/fnut.2023.1179295

**Published:** 2023-06-30

**Authors:** Asmaa M. N. Fatani, Joon Hyuk Suh, Jérémie Auger, Karima M. Alabasi, Yu Wang, Mark S. Segal, Wendy J. Dahl

**Affiliations:** ^1^Department of Food Science and Human Nutrition, University of Florida, Gainesville, FL, United States; ^2^Food and Nutrition Department, King Abdulaziz University, Jeddah, Saudi Arabia; ^3^Rosell Institute for Microbiome and Probiotics, Lallemand Health Solutions, Montréal, QC, Canada; ^4^Foods and Nutrition Department, School of Health Science and Wellness, Northwest Missouri State University, Maryville, MO, United States; ^5^Department of Nephrology, Hypertension and Renal Transplantation, College of Medicine, University of Florida, Gainesville, FL, United States; ^6^North Florida South Georgia VHS, Gainesville, FL, United States

**Keywords:** microbiota, hemodialysis, uremia, fiber, *p*-cresyl sulfate, indoxyl sulfate, stool form, dietary protein

## Abstract

**Background:**

Fiber is a potential therapeutic to suppress microbiota-generated uremic molecules. This study aimed to determine if fiber supplementation decreased serum levels of uremic molecules through the modulation of gut microbiota in adults undergoing hemodialysis.

**Methods:**

A randomized, double-blinded, controlled crossover study was conducted. Following a 1-week baseline, participants consumed muffins with added pea hull fiber (PHF) (15 g/d) and control muffins daily, each for 4 weeks, separated by a 4-week washout. Blood and stool samples were collected per period. Serum *p*-cresyl sulfate (PCS), indoxyl sulfate (IS), phenylacetylglutamine (PAG), and trimethylamine *N*-oxide (TMAO) were quantified by LC–MS/MS, and fecal microbiota profiled by 16S rRNA gene amplicon sequencing and specific taxa of interest by qPCR. QIIME 2 sample-classifier was used to discover unique microbiota profiles due to the consumption of PHF.

**Results:**

Intake of PHF contributed an additional 9 g/d of dietary fiber to the subjects’ diet due to compliance. No significant changes from baseline were observed in serum PCS, IS, PAG, or TMAO, or for the relative quantification of *Akkermansia muciniphila, Faecalibacterium prausnitzii, Bifidobacterium,* or *Roseburia,* taxa considered health-enhancing. Dietary protein intake and IS (*r* = −0.5, *p* = 0.05) and slow transit stool form and PCS (*r* = 0.7, *p* < 0.01) were significantly correlated at baseline. PHF and control periods were not differentiated; however, using machine learning, taxa most distinguishing the microbiota composition during the PHF periods compared to usual diet alone were enriched *Gemmiger*, *Collinsella,* and depleted *Lactobacillus*, *Ruminococcus*, *Coprococcus,* and Mogibacteriaceae.

**Conclusion:**

PHF supplementation did not mitigate serum levels of targeted microbial-generated uremic molecules. Given the high cellulose content, which may be resistant to fermentation, PHF may not exert sufficient effects on microbiota composition to modulate its activity at the dose consumed.

## Introduction

Loss of kidney function leads to an accumulation of uremic molecules, such as *p*-cresyl sulfate (PCS) and indoxyl sulfate (IS), associated with poor quality of life (1) and increased mortality (2). Given that several gut-derived uremic molecules are protein-bound and, thus, dialysis offers limited removal of these toxins, alternative therapies are needed to mitigate uremia. Diet therapy has been proposed, as diets lower in protein and higher in fiber, favoring saccharolytic vs. proteolytic fermentation in the gut (3), mitigate the production of the microbially generated metabolites contributing to uremia (4–6). Whereas protein restriction may diminish uremic toxin levels in the earlier stages of chronic kidney disease (CKD) (7), this is not a viable therapeutic option for the dialysis population due to their increased protein needs (8) and high risk of malnutrition (9). Enhancing fiber intake may be a potential dietary therapy to reduce uremia.

Plant-based diets, providing plenty of diverse fiber substrates for gut microbiota, are increasingly being recommended for the CKD population (10, 11) for the mitigation of inflammation (12, 13) and the reduction of uremic symptoms (14). Although a vegetarian diet has been shown to reduce uremic toxin levels (15), implementing such plant-based diets in the dialysis population is met with the challenges of poor appetite (16–18), food preferences (19), perceptions of dietary restrictions (20, 21), food insecurity (22) and in some cases limited access to fresh foods (23). Additionally, recommending plant-based diets may exacerbate the time, convenience, and financial barriers to dietary change experienced by hemodialysis patients (24). Isolated fibers, which can be easily incorporated into common foods, particularly baked goods, offer alternative sources of fiber for the dialysis population without significant additions of potassium, phosphorus, and sodium – minerals of concern.

Prebiotic fibers, at 9–20 g/day, have shown efficacy for reducing blood levels of uremic toxins such as PCS in some studies (25, 26) but not in others (27, 28). However, highly fermentable prebiotic fibers such as inulin and fructooligosaccharides may contribute to gastrointestinal symptoms (27, 29), mitigate appetite, and enhance satiety (30), which may contribute to the risk of malnutrition in the dialysis population. In contrast, fibers that are resistant to fermentation, such as hull and bran fibers (31) or which are more slowly fermented, such as resistant starches (32), may be better tolerated while still reducing uremia. However, there are limited data to support the efficacy of resistant starch in reducing uremic toxins (33), and intact bran or hull fibers have been unexplored in dialysis patients.

Pea hull fiber (PHF), the finely-ground outer hulls of yellow field peas, is rich in dietary fiber, and bioactive components, including polyphenols (34). PHF, a non-viscous fiber ingredient, consists of soluble and insoluble fibers, primarily cellulose (65%) and pectin (16%), with oligosaccharides, hemicelluloses, and lignin as minor components (34), a profile that suggests reduced fermentability compared to typical prebiotics. However, in older adults, PHF has been shown to modulate fecal microbiota without appetite suppression (35), suggesting it may be fermentable and also an appropriate fiber for hemodialysis patients at nutritional risk. PHF at 10 g/d, in combination with 15 g of inulin, led to lower blood levels of PCS in adults with stage 3 chronic kidney disease (36). PHF may elicit uremic suppressing effects through saccharolytic fermentation if microbially accessible or, if resistent, may decrease colonic transit time through stool bulking, as do bran fibers (37), thereby lessening proteolytic fermentation (38). The primary aim of this study was to determine the effect of PHF supplementation on serum levels of PCS in adults undergoing hemodialysis. The secondary aims were to determine the effect of PHF supplementation on serum levels of IS, phenylacetylglutamine (PAG), and trimethylamine *N*-oxide (TMAO), and fecal microbiota composition. Additionally, to monitor the safety and tolerance to PHF supplementation in the hemodialysis patient population, blood chemistry, gastrointestinal function and symptoms, inflammatory markers, dietary intake, appetite, and quality of life were monitored.

## Materials and methods

### Study design

**Figure 1:**
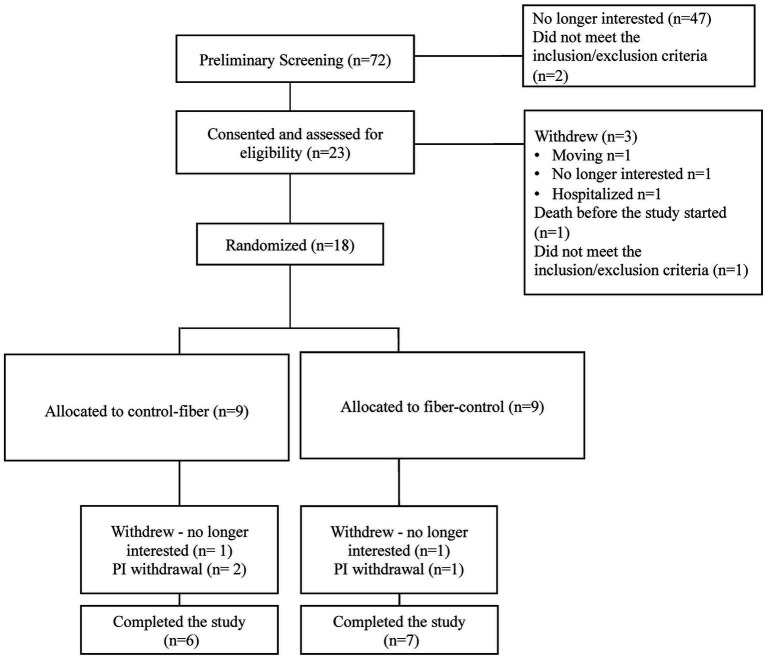
Participant recruitment, randomization, and study completion. Principal Investigator (PI) withdrawals due to hospitalizations.

A 13-week, randomized, double-blind, controlled crossover study was conducted in adults undergoing hemodialysis and included a one-week baseline, two 4-week interventions (PHF and control), and a 4-week washout period between interventions (Figure 1). At the beginning of each period, pre-dialysis blood and fecal samples were collected, and quality of life, weight, blood pressure, and dietary intake were monitored. Height, body composition, and hand-grip strength were measured at baseline. The study protocol was approved by the Institutional Review Board of the University of Florida (IRB201701457), and the trial was registered at ClinicalTrials.gov (NCT03354364). Written informed consent was obtained from all study participants, and study procedures were in accordance with the Declaration of Helsinki.

### Participants

Adults undergoing hemodialysis were recruited from a single center in Florida, United States. Participants were excluded for food allergy, lactation, pregnancy, previous or current treatment for any gastrointestinal disease (gastric ulcers, Crohn’s disease, celiac disease, ulcerative colitis, etc.), use of medications for diarrhea or constipation, or non-continuation of any probiotic supplements.

### Randomization and intervention

An unaffiliated researcher completed the randomization scheme. Randomization was performed by a sealed envelope method; the study coordinator opened a sealed envelope containing the allocated treatment regimen at the time of randomization.

### Study foods

PHF (Best Cooking Pulses; Portage la Prairie, MB, Canada; total dietary fiber 92%; insoluble 85%, soluble 7%) was added to vanilla, lemon, cinnamon, and chocolate chip mini muffins with each muffin providing 5 g of PHF and 70 kcal. A sensory evaluation panel was carried out to test the acceptability of the chocolate chip muffin. Both muffins were rated acceptable; however, the control muffin rated higher for overall liking and texture liking, with no differences for flavor, sweetness liking, or dryness (39). Participants were provided frozen muffins every 2 weeks, asked to thaw and consume three muffins daily (providing ~15 g/day of PHF), and to return any uneaten muffins. Control muffins, also consumed at 3 per day, provided 0.9 g of fiber and 67 kcal per muffin. A daily question in the participants’ study diary assessed compliance with muffin intake for the entirety of the intervention period.

### Clinical assessments

Post- and pre-dialysis weight in duplicate were measured at each visit, and height was measured at baseline using a flat scale and portable stadiometer (Seca® models 217 and 874, Mount Pleasant, SC), respectively. Blood pressure was measured at each visit using the monitor connected to the hemodialysis machine. At baseline, post-dialysis, hand-grip strength was assessed using the Jamar^®^ Plus+ digital dynamometer (Patterson Medical, Warrenville, IL, United States) (40) and body composition by Bioelectrical Impedance Analysis Xitron Hydra ECF/ICF 4200 equipment (Xitron Technologies, San Diego, CA, United States). In their study diary, participants recorded their daily stool frequency and stool form using Bristol Stool Form Scale (BSFS) (41). Gastrointestinal symptoms were assessed weekly using the Gastrointestinal Symptom Response Scale (GSRS) (42). The Simplified Nutritional Appetite Questionnaire (SNAQ) (43) was assessed weekly. A SNAQ score of >14 of 20 points indicates no risk of weight loss, whereas a score of 14 or less indicates a significant risk of at least 5% weight loss within 6 months. During each period, the KDQOL-36 with its five subscales (Physical Composite, Mental Composite, Burden of Kidney Disease, Symptoms and Problems of Kidney Disease, and Effects of Kidney Disease) was administered, and the total score was calculated using the standardized formulae (range from 0 to 100), with a higher score indicating a better health-related quality of life (44).

Pre-dialysis blood samples were collected at baseline and on the first day of each period via the hemodialysis vascular access. The components of a comprehensive metabolic profile were analyzed by standard laboratory methods. TNFα, IL-6, and IL-10 were measured using the Bio-Plex Pro™ Human Cytokine Standard 27-plex, Group 1 (Lot #64103331) (Bio-Rad Laboratories, Hercules, CA, United States) with the Bio-Plex 200 suspension array system (Bio-Rad Laboratories) and Bio-Plex Manager software (v6.2), according to the manufacturer’s instructions. Serum samples were thawed at 4°C overnight and then vortexed and centrifuged; the collected supernatants were transferred into new 1.5 mL tubes. The samples were diluted at 1:4 by using the diluent provided with the kit. The Quick Guide supplied with the kit was followed to finish running the assay.

### Dietary assessment

A team of trained dietetic graduate students assessed dietary intake at baseline and the final week of each period. Participants were typically interviewed for their 24-h dietary recalls on dialysis days, and thus intake generally reflected non-dialysis days. Three 24-h recalls for each period were analyzed for nutrition composition using Food Processor Nutrition Analysis Software (ESHA version 11.3.2). Diet quality at baseline was assessed using the Healthy Eating Index (HEI-2015), which applies the recommendations of the 2015–2020 Dietary Guidelines for Americans as previously described (45).

### Quantification of uremic metabolites

The uremic molecules PCS, IS, PAG, and TMAO levels were measured, as were trimethylamine (TMA) and carnitine, to elucidate TMAO metabolism. Ten mL Vacutainer® Plus SST™ serum separation tubes were centrifuged for 10 min at 4°C in a Hettich Instruments ROTINA 420R centrifuge at 800 x g (1,500 rpm). Serum was aliquoted in 250 μL tubes and stored at −80°C until analyzed. PCS and IS were obtained from Alsachim (Illkirch-Graffenstaden, France), and PAG was sourced from LGC (Middlesex, United Kingdom). Carnitine, trimethylamine hydrochloride, TMAO, _L_-carnitine-methyl-d_3_ hydrochloride (carnitine-d_3_), trimethylamine-d_9_-*N*-oxide (TMAO-d_9_), and *p*-toluene sulfonic acid sodium salt were sourced from Sigma-Aldrich (St. Louis, MO, United States). Trimethylamine-d_9_ hydrochloride (TMA-d_9_) was obtained from Toronto Research Chemicals Inc. (North York, ON, Canada). Acetonitrile, water, ammonium formate, and formic acid were sourced from Fisher Scientific (Fair Lawn, NJ, United States) and were of liquid chromatography-mass spectrometry (LC–MS) grade. For standard solutions preparation, stock solutions of individual analytes and internal standards were prepared at a concentration of 1,000 μg/mL in acetonitrile or water-acetonitrile (1:1) and stored at −80°C until used for analysis. Standard mixture solutions used for calibration curves were prepared as follows: for PCS, IS, and PAG analysis, diluting concentrated solutions with water-acetonitrile (1:1), and for carnitine, TMA, and TMAO analysis, diluting concentrated solutions with just acetonitrile.

Following standard preparation, 20 microliters of serum were aliquoted into a 1.5 mL Eppendorf tube and mixed with acetonitrile that contained internal standards (1 μg/mL of *p*-toluene sulfonic acid, 0.75 μg/mL of carnitine-d_3_, 0.5 μg/mL of TMA-d_9_, and 0.15 μg/mL of TMAO-d_9_) to reach a total of 1 mL. Samples were then vigorously vortexed for 10 min, followed by sample centrifuging at 25000 g at 4°C for 5 min. After centrifugation, the supernatants for carnitine, TMA, and TMAO were directly injected into a liquid chromatography with tandem mass spectrometry (LC–MS/MS) system, while the supernatants for PCS, IS, and PAG were first diluted with the same volume of water before they were injected into the LC–MS/MS system. The individual samples were extracted in triplicates (*n* = 3).

The PCS, IS, and PAG quantified in serum were done by LC–MS/MS analyses, applying an Ultimate 3,000 LC system coupled to a TSQ Quantiva triple quadrupole mass spectrometer (Thermo Fisher Scientific, San Jose, CA, United States). Separation of PCS, IS, and PAG was performed using a Waters Acquity BEH C18 column (2.1 × 50 mm, particle size 1.7 μm) at a column temperature of 25°C using a gradient elution with 0.1% formic acid in water (eluent A) and 0.1% formic acid in acetonitrile (eluent B). The gradient program was set as follows: 0–3 min 10–50% B, 3–4 min 50–95%, and 4–6 min 95% B. The column was re-equilibrated in 3.5 min using the initial composition of the mobile phase. The injection volume was 2 μL with a flow rate of 0.2 mL/min. The mass spectrometer was equipped with an electrospray ionization (ESI) interface, operating in both positive and negative ionization modes. The spray voltage was set as 3,500 V for positive mode and 2,500 V for negative mode. Other ESI parameters were set as follows: ion transfer tube temperature, 325°C; vaporizer temperature, 275°C; sheath gas, 35 Arb; aux gas, 10 Arb; and sweep gas, 0 Arb. Selective reaction monitoring mode was used when the MS/MS detection was operated. Dwell time was 100 msec, and CID gas was set at 2 mTorr. MS/MS parameters for each analyte (PCS, IS, and PAG) were optimized using flow injection analysis of individual standards. Xcalibur software (Ver. 3.0) was utilized for data processing and instrument control.

Carnitine, TMA, and TMAO were analyzed using the same LC–MS/MS described above, with a different chromatographic system (hydrophilic interaction liquid chromatography, HILIC). These compounds were separated using a Thermo scientific Accucore HILIC column (2.1 × 100 mm, particle size 2.6 μm). The column temperature was maintained at 30°C using gradient elution with 15 mM ammonium formate (pH 3.5) (eluent A) and acetonitrile (eluent B). The gradient program was set as follows: 0–4 min 90–60% B and 4–6 min 60% B. The initial composition of the mobile phase was used to re-equilibrate the column in 4 min. The injection volume was 2 μL with a flow rate of 0.4 mL/min. The mass spectrometer was equipped with an ESI interface, operating in the positive ionization mode. The positive spray voltage was 3,500 V, and the ion transfer tube temperature was maintained at 340°C. The other ESI parameters were set as follows: vaporizer temperature, 350°C; sheath gas, 45 Arb; aux gas, 15 Arb; and sweep gas, 1 Arb. The selective reaction monitoring mode was used to operate the MS/MS detection. Dwell time was 100 msec, and CID gas was set at 2 mTorr. MS/MS parameters for every analyte were adjusted using flow injection analysis of individual standards. Xcalibur software (Ver. 3.0) was used for data processing and instrument control.

### Microbiota analyses

Stool collections were made using a plastic container with a lid (Fisherbrand™ Commode Specimen Collection System) during the baseline and the last week of each period. Upon receipt, one sample was homogenized for microbiota analysis, aliquoted into 5 mL tubes, and stored at −80°C. Total DNA was isolated from the 250–350 mg homogenized stool sample, as previously described (46). The QIAamp^®^ Fast DNA Stool Mini Kit (Qiagen, Hilden, Germany) was used, and the manufacturer’s instructions were followed with the following modifications. Sodium phosphate buffer as two 50 mmol/L was utilized for washing preceding the addition of InhibitEX (Qiagen) and a 0.1-mm zirconia/silica bead beating step (~250–350 mg/tube, 4 m/s for 1 min × 3) following the incubation with InhibitEx. Then, Nanodrop was used to determine the concentration of the DNA. Samples were stored at −20°C until additional analysis. Before the process of qPCR analysis, molecular biology-grade water was used to dilute the samples fivefold.

A previously reported method was followed to analyze gut microbiota composition and diversity (47). In brief, gene-specific primers for the V4 hypervariable region of the 16S ribosomal RNA gene were used for DNA amplification and tagged with unique identifiers. As previously described (44), for the bacterial 16S ribosomal RNA gene libraries preparation, Illumina’s“16S Metagenomic Sequencing Library Preparation” guide (part #15044223 Rev. B) was used. The Qiagen HotStarTaq MasterMix was utilized for the first PCR (amplicon PCR) at 25 cycles with heat at temperatures of 55°C. However, only 50% of the reagent volumes were used for the second (index PCR) PCR. The template-specific primers, 515f (5´-GTGCCAGCMGCCGCGGTAA-3´) and 806R (5´-GGACTACHVGGGTWTCTAAT-3´), flanked with appropriate overhang adapter sequences, were utilized. Samples were diluted, pooled, and sequenced using a 500-cycle MiSeq Reagent Kit v3 before loading on an Illumina MiSeq.

Taxonomic attribution of the amplicon sequence variants (ASVs) was used to generate the taxonomic profiles for each sample using the QIIME^™^ 2 feature-classifier machine learning-based tool and the database, GreenGenes (47). The taxonomic profiles for each participant in each period were generated on individual taxa and strains. Principal coordinate analysis (PcoA), weighted and unweighted UniFrac, alpha diversity (evenness and faith) profiles, and individual taxonomic profiles were produced and assessed for each participant in each period using QIIME^™^’s visualization tools. QIIME^™^ 2 sample-classifier was used to discover any unique microbiota profile that may be determined because of PHF consumption and distinguish the taxa differing in abundance. For each participant, both treatments were compared to the baseline or washout, depending on the period before the treatment.

Given their positive associations with health (48–51), relative quantification of *Akkermansia muciniphila*, *Faecalibacterium prausnitzii, Bifidobacterium,* and *Roseburia* was performed. 16S ribosomal DNA Universal Bacterial primers were used for DNA normalization. The 2^-∆∆CT^ algorithm method was used to analyze the changes in the relative fold gene expression when comparing baseline to the other study periods. The *A. muciniphila* and *F. prausnitzii* primer sequences and assay conditions were obtained from previous studies (52, 53). However, the primers for *Bifidobacterium* were designed in-house as described previously (forward: TGG AAG GTC TCG ATG GAG GT and reverse: CTG GAC AAG CCG TTC CTG AT) and utilized the same assay and cycling conditions as listed for the primers used in strain quantification. A dissociation curve analysis (60°C to 95°C) was also performed to ensure primer specificity for all assays. The epMotion5075tc liquid handling robot and Select SYBR Mastermix (Thermo Fisher Scientific) were used for all qPCR reaction preparations. The analysis was performed on the CFX384 Touch Real-Time PCR Detection System (Bio-Rad Laboratories).

### Statistical analysis

The sample size was calculated based on expected PCS change using data from our previous study of fiber supplementation in adults with CKD (54). The expected PCS mean difference was 4 mg/L with a standard deviation of 6 mg/L. Assuming an alpha = 0.05 and power = 0.80 (80%), a sample of 21 was needed. To account for an expected 20% dropout rate, 25 participants were targeted.

Linear mixed models with fixed factors treatment (PHF, Control, Baseline, Washout), sequence (PHF-Control, Control-PHF), and the interaction between the two was used. The interaction tests were for order effects of treatment. The random effects in the model included random ID (participant) and an auto-regressive correlation structure. For uremic molecules analysis, the data were log-transformed for analysis, and variance was allowed to differ between time points. Additional analysis was conducted to investigate the association between some variables and uremic molecules using the Pearson correlation coefficient test. Alpha was set at 0.05. Data are presented as mean ± SE unless otherwise indicated.

Individual symptoms evaluated by the GSRS were averaged into syndrome scores. For the GSRS symptoms analysis, the data were log-transformed for analysis and variance. For BSFS analysis, data were divided into three categories to detect the transit time, following a previously published method (55). Stool forms were grouped as slow transit, types 1 and 2, normal transit, types 3, 4, and 5, and fast transit, types 6 and 7. The frequency procedure was used to detect the frequency of each transit type during the whole study period. Then, the GIMMIX procedure was conducted to examine the effect of treatments on transit time.

The Mann–Whitney test was performed to compare the relative quantification of each treatment group for each type of bacteria (*A. muciniphila, F. prausnitzii, Bifidobacterium,* and *Roseburia*). Kruskal-Wallis test was used to determine the differences between treatments for evenness α-diversity and faith α-diversity. The Quantitative Insight Into Microbial Ecology-2 (QIIME 2) software suite was used as described previously (56) to determine the results for α-diversity and principal component analysis (weighted and unweighted UniFrac). A machine learning model was used to predict PHF vs. control or usual diet (baseline/washout). The accuracy of the results of the Sample Classifier was visualized using a confusion matrix (as it is more informative than the Accuracy Ratio alone), showing the prediction made on the Test Dataset (a subset of ~1/3 of the full dataset) after algorithmic training on the Training Dataset.

## Results

### Study population

**Figure 2:**
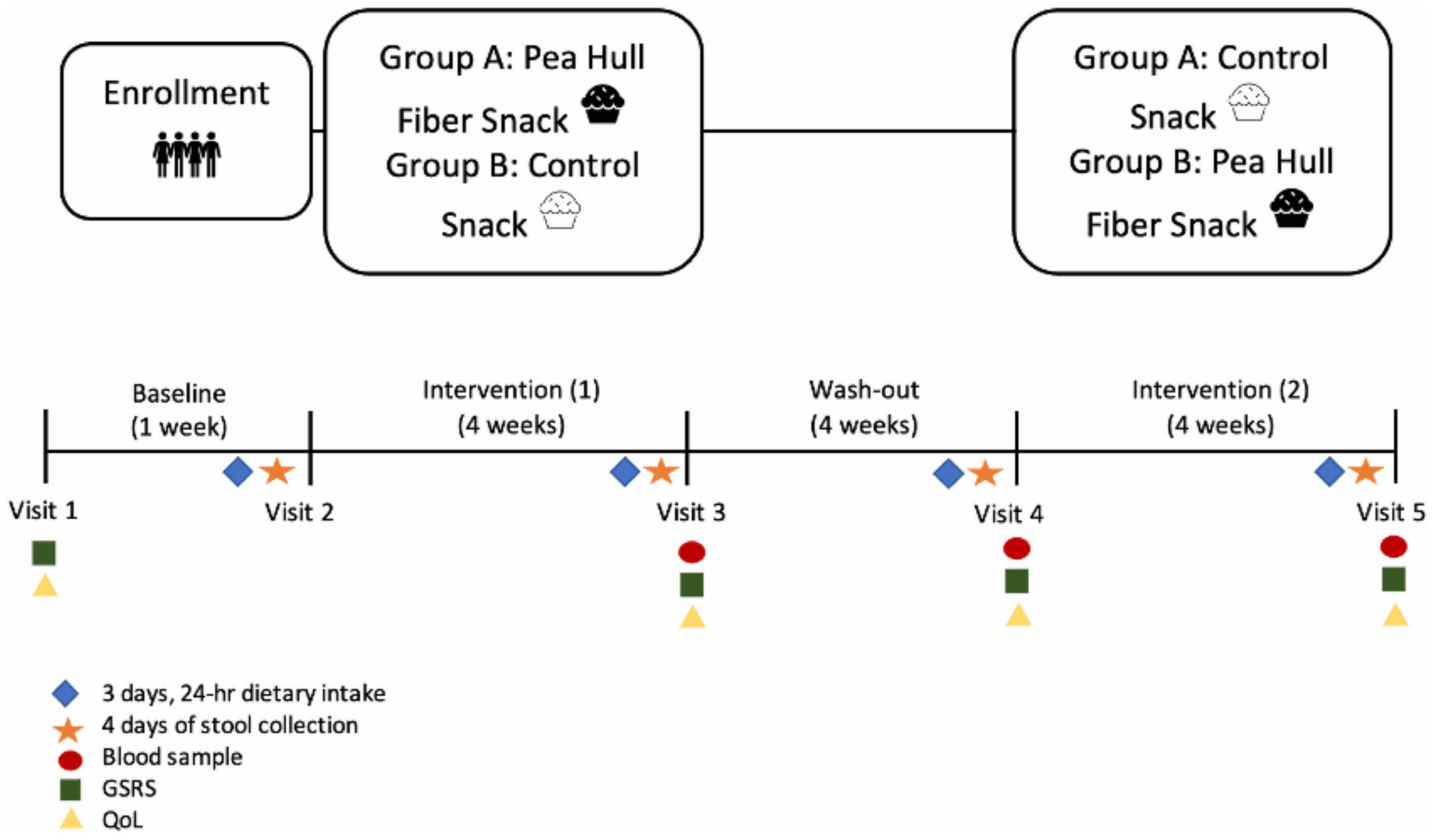
Study design for the 13-week double-blinded, randomized, crossover trial investigating the effects of pea hull fiber (PHF) on uremic toxins. GSRS: Gastrointestinal Symptoms Rating Scale. QoL: Kidney Disease Quality of Life 36-Item Short-Form Survey.

From December 2017 to January 2019, 23 adults undergoing hemodialysis were recruited from a single hemodialysis center in Florida, United States. The study flow is shown in Figure 2. Post-consent inclusion/exclusion criteria excluded one participant; one died before the initiation of the intervention, five participants withdrew, and the principal investigator withdrew three due to hospitalizations. Table 1 presents the baseline characteristics of the study participants. Most participants were non-Hispanic Black and had been undergoing hemodialysis for more than 2 years. Compliance with the interventions was 61.9% for PHF (+ 9 g/d fiber) and 81.6% for the control muffins.

**Table 1:** Demographic characteristics and biochemical data of participants receiving hemodialysis.

Characteristics		Biochemical	
Gender M/F, *n*	10/8	Albumin, g/dL	4.0 ± 0.1
Age, y, median (range)	52 (21–71)	Albumin/Globulin, (calc)	1.3 ± 0.1
Race, *n* (%)		Anion Gap, mmol/L	130.1 ± 12.2
African American	12 (67)	BUN/Creatinine ratio, (calc)	5.9 ± 0.4
Asian	0	Calcium, mg/dL	9.1 ± 0.2
White	4 (22)	Carbon dioxide, mmol/L	23.4 ± 0.7
More than one race	2 (11)	Chloride, mmol/L	97.6 ± 0.7
Ethnicity, *n* (%)		Creatinine, mg/dL	9.6 ± 0.7
Hispanic	0	eGFR, ml/min/1.73M^2^	6.6 ± 0.7
Non-Hispanic	18 (100)	Glucose, mg/dL	139.8 ± 22.3
Dialysis, *n* (%)		Magnesium, mg/dL	2.3 ± 0.1
<1 y	3(17)	Phosphorus, mg/dL	5.7 ± 0.4
1–2 y	2 (11)	Potassium, mmol/L	4.7 ± 0.2
>2 y	13 (72)	Sodium, mmol/L	136.9 ± 0.7
Body Mass Index (BMI), *n* (%)		Total protein, g/dL	7.4 ± 0.2
Underweight (<18.5)	2 (11)	Urea nitrogen, mg/dL	55.0 ± 3.6
Normal (18.5 to 24.9)	8 (44)		
Overweight (25 to 29.9)	3 (17)	Cytokines	
Obese (>30)	5 (28)	IL-10, pg./mL	3.3 ± 1.8
Body Fat (%) mean ± SE	26.4 ± 3.0	IL-6, pg./mL	5.9 ± 1.1
Hand-grip strength, (kg) mean ± SE	31.3 ± 2.7	TNF-α, pg./mL	130.1 ± 14.3

### Clinical assessments

Clinical assessments were generally unaffected by the interventions. The baseline hand-grip strength is reported in Table 1. According to the Matos et al. (57) criteria, 27% of study participants exhibited hand-grip strength below the cut point for a higher risk of death, and another 20% fell below the cut point for increased risk of frailty (58). According to the SNAQ score, 67% of the participants were at no risk of weight loss, whereas 33% were at high risk.

Baseline stool frequency (Table 1) was reported at 5.2 ± 0.6 stools per week and differed from PHF (9.8 ± 1.3), control (10.2 ± 1.4), and washout (10.0 ± 1.2) periods (*p* < 0.01). Stool forms at baseline were 30.2% slow transit (BSFS 1 and 2), 39.7% normal transit (BSFS 3–5), and 30.1% fast transit (BSFS 6 and 7) and did not differ with interventions. The baseline GSRS syndrome scores of abdominal pain (1.5 ± 0.2), reflux (1.6 ± 0.2), indigestion (2.0 ± 0.2), diarrhea (1.9 ± 0.2), constipation (1.9 ± 0.3) were unchanged with interventions.

The KDQOL-36 components at baseline (Table 1) of SF-12 Physical Composite (35.1 ± 2.3), SF-12 Mental Composite (50.2 ± 3.1), KDQOL-36 Burden of Kidney Disease (45.5 ± 6.6), KDQOL-36 Symptoms and Problems of Kidney Disease (77.2 ± 3.8), and KDQOL-36 Effects of Kidney Disease (70.8 ± 5.1) showed no differences between periods (data not shown).

Biochemical measurements of the complete metabolic panel at baseline are shown in Table 1; there were no differences between periods (data not shown). Of the targeted cytokines (Table 1), there were no significant differences between study periods for TNF-α. However, for IL-6, the washout was higher compared to other study periods. Very low, out-of-range values for IL-10 precluded statistical analysis.

### Dietary intake

Energy, macronutrient, and select mineral intakes of the background diet during each period, shown in Supplementary Table S1, remain unchanged from baseline. At baseline, the mean energy intake of study participants was 1786 ± 143 kcal providing 23.8 kcal/kg body weight. Protein intake at 68 ± 7 g/day (0.9 g/kg body weight) and fiber at 11 ± 1 g/day (6.2 g/1000 kcal) provided a protein-to-fiber ratio of 6 to 1. This ratio was reduced to 3.2 to 1 during the PHF period. Carbohydrate intake averaged 224 ± 19 g/d and total fat, 67 ± 7 g/day. The participants’ background diet at baseline was approximately 50% carbohydrate, 15% protein, and 34% fat and showed no significant changes during the study. The mean HEI-15 score was 44.2 ± 2.5 out of the maximum score of 100, indicating poor diet quality. Due to compliance issues, consumption of the study muffins contributed 130 kcal/day during the PHF period and 161 kcal/day during the control period.

### Uremic molecules

The results for PCS, the primary outcome, and IS, PAG, and TMAO are presented in Table 2. No significant differences were observed in serum levels of these uremic molecules (individual data pre and post-PHF are shown in Supplementary Figure S1) nor for TMA or carnitine (data not shown). At baseline, there was a negative correlation between protein intake and serum IS level (*r* = −0.47 and *p* = 0.05) and a positive correlation between the number of slow transit stools, as assessed by BSFS, and serum PCS level (*r* = 0.68 and *p* < 0.01).

**Table 2:** Serum uremic molecules by period for participants receiving hemodialysis.

Metabolite (μmol/L)	Baseline	Fiber	Control	Washout
*p-*Cresyl sulfate	3256.3 ± 505.3	3309.7 ± 668.5	2858.8 ± 464.2	3365.5 ± 558.6
Indoxyl sulfate	166.0 ± 23.4	185.4 ± 26.3	148.8 ± 20.8	166.7 ± 27.4
Phenylacetylglutamine	36.4 ± 5.8	33.6 ± 8.1	43.1 ± 8.0	51.2 ± 11.0
Trimethylamine-*N*-oxide	95.9 ± 12.2	128.3 ± 18.7	140.2 ± 52.8	100.0 ± 13.3

Data presented as mean ± SE.

### Microbiota composition

**Figure 3:**
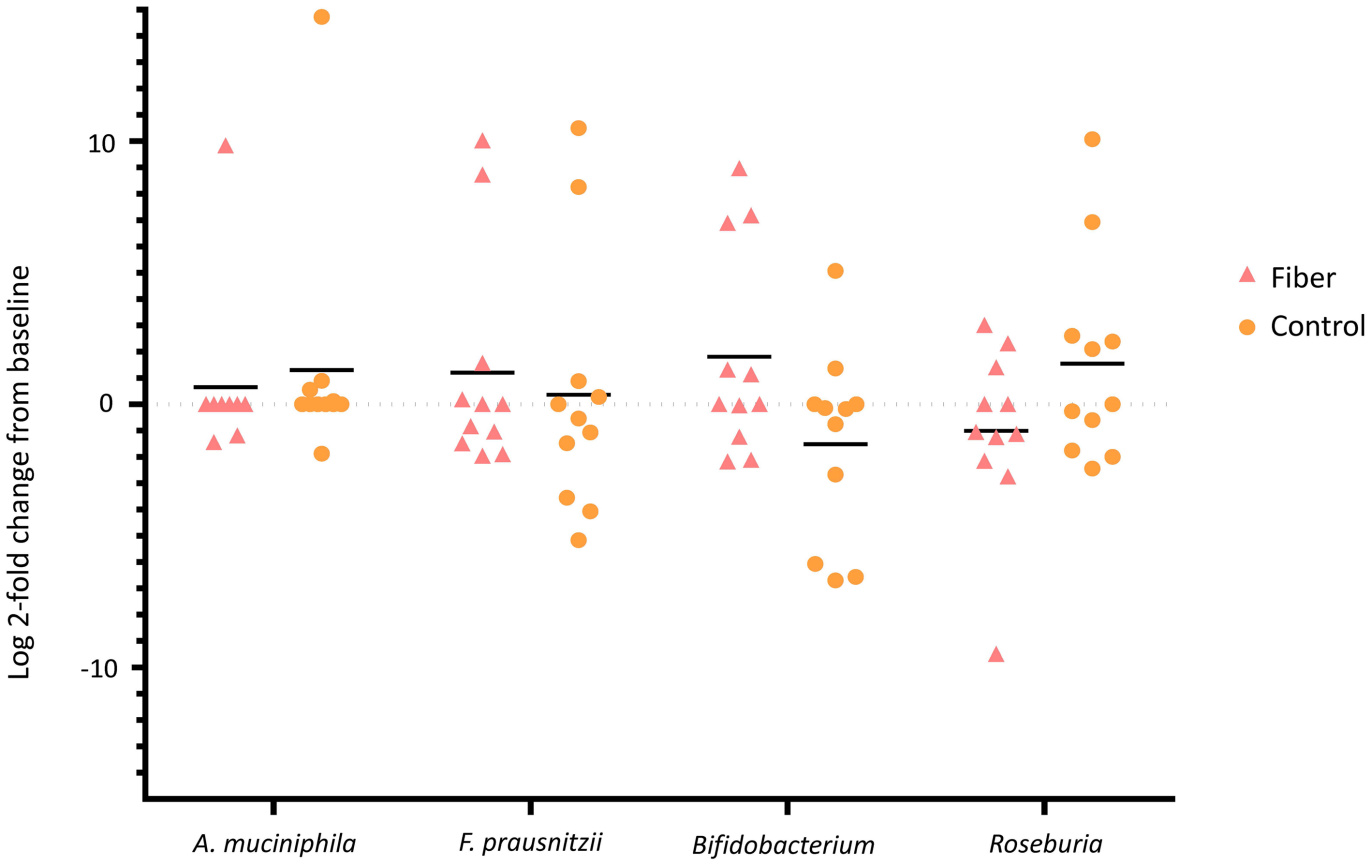
Comparison of the relative fold change of the relative quantification by qPCR of *Akkermansia muciniphila*, *Faecalibacterium prausnitzii*, *Bifidobacterium* spp., and *Roseburia* spp. of fecal samples from participants receiving hemodialysis during the pea hull fiber (Fiber) and control periods. No statistically significant differences were observed.

At the genus level, the relative abundance was highest for *Bacteroides* but was very high (30 to 50%) for some participants and near 0% for others (Supplementary Figure S2). No significant differences in alpha diversity or evenness were seen between PHF and control periods (Supplementary Figure S3), nor between treatments for the relative fold change in the relative quantification of *A. muciniphila, F. prausnitzii, Bifidobacterium,* and *Roseburia* by qPCR (Figure 3).

The weighted and unweighted UniFrac PCoA from QIIME™ of the hemodialysis participants’ datasets visualized using Emperor according to treatment period showed significant overlap (Supplementary Figure S4). PHF may have influenced the microbiota composition profiles. The confusion matrix showed strong accuracy scores on the main diagonal and a good accuracy result of 83% for the control and an accuracy of 100% for the PHF predictions alone compared to baseline/washout periods. Only six important taxa were used to predict PHF compared to participants’ usual diet (baseline + washout), whereas 49 taxa predicted control. PiratePlots of the relative abundance of the six important taxa that predicted PHF consumption include *Coprococcus*, *Lactobacillus*, *Ruminococcus*, *Gemmiger*, *Collinsella*, and the family, Mogibacteriaceae; only *Gemmiger* and *Collinsella* showed higher abundance (Supplementary Figure S5). The algorithm using the QIIME 2 classifier was unable to differentiate between fiber and control; the prediction was lower than by chance. Similarly, no differentiation was seen for intervention (fiber + control) compared to usual diet intake (baseline/washout), suggesting no ‘muffin’ effect. Of note, no participants reported being prescribed an antibiotic during the study.

## Discussion

The study findings confirm that adults undergoing hemodialysis have inadequate intakes of dietary fiber, in agreement with previous reports (59–62). This fiber deficit may be in part due to long-standing dietary recommendations to restrict plant foods higher in phosphorus (whole grains and legumes) and potassium (legumes, fruits, and vegetables). As assessed by the HEI-2015, study participants consumed almost exclusively refined grains and had very low intakes of fruit, a dietary pattern contributing to their dietary fiber deficit. Given the questionable effectiveness of restricting plant foods, with their lower phosphorus bioavailability, to manage serum phosphorus levels (63), a concerted effort may be needed to enhance diet quality, particularly regarding whole-grain intake, and thus increase dietary fiber intake of hemodialysis patients and the CKD population in general. With many lower potassium options available, enhanced fruit and vegetable intake also is needed to improve fiber intake in this population (64). However, as low socioeconomic status is associated with low fruit and vegetable consumption (65) and end-stage kidney disease (66), this may be a challenging long-term approach. In the short term, recommending the replacement of refined grain-based foods with sensorily acceptable, fiber-fortified versions may be a feasible approach to improving the fiber intake of the hemodialysis patient population.

Although in the US, there is no clinical practice guideline for fiber intake for adults undergoing hemodialysis (8), their low fiber intake strongly suggests a lack of microbial-available carbohydrate substrate in the colon, which facilitates uremic molecule generation, particularly in an environment of reduced protein digestion (67). Although the PHF intervention enhanced fiber intake by approximately 9 g/day and improved the protein-to-fiber ratio from 6:1 to 3:1, PHF was ineffective in suppressing serum levels of targeted uremic toxins. Additionally, inflammatory cytokines remained unchanged, although higher dietary fiber intake is associated with less inflammation in individuals with CKD (68, 69). Reported reduced serum PCS with an escalating dose of 20 g/d of oligofructose-enriched inulin in an open-label study in hemodialysis patients (26), had suggested that the prebiotic inulin may be a driver of uremic molecule reduction. However, Poesen et al. (28) showed no effects on the serum levels of PCS, *p*-cresol glucuronide, IS, or PAG in adults with CKD after 4 weeks of 20 g/day of the prebiotic arabinoxylan oligosaccharides, even though in healthy subjects, arabinoxylan oligosaccharides decreased urinary PCS excretion after only 2 weeks of supplementation (70). Similarly, an intervention of 12 g/day of fructooligosaccharides showed no significant effects on serum or urinary PCS in non-dialysis-dependent adults with CKD (71), nor did 10–15 g/day inulin supplementation in hemodialysis patients (27). Alternatively, participants’ protein intake, with its expected incomplete digestion (67), may have contributed more to uremic molecule levels by specifically providing aromatic amino acid substrates. Surprisingly, baseline protein intake was not associated with serum PCS or PAG, and a negative correlation was seen for IS.

BSFS of 1 and 2 comprised more than 30% of the reported stool forms suggesting a significant level of slow gut transit in this patient population. Examining individual data, the number of slow transit stools per week was associated with the serum levels of PCS. This finding is in agreement with Ramos et al. (72), who reported that a BSFS score of <3 was associated with higher serum PCS but not IS in a cross-sectional analysis of CKD patients. Similarly, Pereira et al. (73) reported that peritoneal dialysis patients with a BSFS of <3 had higher levels of total and free PCS and indole-3-acetic acid but not IS. Indeed, slow transit time, which depletes microbially available carbohydrates, may contribute to proteolytic fermentation and PCS production. As insoluble, less fermentable fiber sources such as wheat bran have been shown to mitigate slow transit time (74), interventions with adequate doses of such fibers may decrease PCS. PHF, although an insoluble hull fiber, did not modulate BSFS. This may be due to its fine particle size, which may have enhanced fermentability, an effect shown with wheat brans (75), thereby reducing its potential impact on transit time. Low consumption of fruit may have contributed to the high percentage of slow transit stool forms, as increased fruit intake has been associated with a lower risk of constipation in adults receiving hemodialysis (76).

It is well established that dysbiosis of CKD is worsened by hemodialysis (77). In the present study, relative abundance was highest for the genus *Bacteroides*. Similarly, *Bacteroides* was the most abundant genus in Chinese (78) and Taiwanese (79) hemodialysis patients, specifically *Bacteroides ovatus*, *Bacteroides caccae*, and *Bacteroides uniformis* in the latter study. PHF supplementation did not modulate relative fold changes of *A. muciniphila, F. prausnitzii, Bifidobacterium,* and *Roseburia,* taxa considered health enhancing (48–51). However, the machine learning model confusion matrix showed strong accuracy scores, and the relative abundance of *Coprococcus*, *Lactobacillus*, *Ruminococcus*, *Gemmiger*, *Collinsella*, and Mogibacteriaceae predicted PHF. Of note, *Coprococcus* showed higher relative bacterial proportions by LDA effect size (LefSe) comparison (as well as *Methanobrevibacter* and Peptostreptococcaceae) with PHF supplementation in a subgroup of older adults who exhibited increased gastrointestinal symptoms, a proxy for fermentation, within 2 weeks of PHF consumption (35). In contrast, a 12-week study of adults with overweight or obesity showed that PHF decreased the relative abundance *of Actinomyces*, *Holdermania,* and *Oscillospira,* and increased *Lachnospira* over time, the latter a possible outcome confounded by weight loss (80). Although antibiotic use was not an exclusion criterion, none was reported during the trial and therefore did not affect outcomes. In summary, there is insufficient evidence to support that PHF exerts a consistent effect on fecal microbiota composition.

A notable limitation of this study was the high withdrawal and dropout rate. Given the high levels of morbidity in the hemodialysis population, a higher level of over-sampling is needed for future studies. An additional limitation was the less-than-optimal compliance with study snacks consumption, which may have been due to the lower acceptability of the PHF muffins compared to the control muffins; some participants commented that the muffins were somewhat dry. Furthermore, providing a variety of fiber-fortified foods may lead to better compliance, as previously demonstrated (54). Low adherence may also have been due to poor appetite commonly experienced by individuals receiving dialysis. Additionally, given that dysbiosis is exhibited in hemodialysis patients, a more extended intervention period may be needed to modulate fecal microbiota and, thus, serum levels of uremic toxins.

Although fiber fortification with PHF did not mitigate uremic molecule generation, higher doses or supplementation with mixed sources of fiber may demonstrate efficacy. More aggressive dietary interventions may be needed to effect changes in the microbiota composition and activity. However, adherence to a healthful Mediterranean dietary pattern failed to explain PCS and IS levels in a CKD cohort (81). Fecal microbiota transplantation (82) and intensive intestinal interventions (83) have been proposed as therapies for uremia toxin reduction but lack practicality. Alternatively, select targeting of microbes contributing to uremic toxins generation requires exploration. However, research is first needed to elucidate the relationships between the microbiome and uremic toxins levels, given the dramatic between-subject variations observed in hemodialysis patients.

## Data availability statement

The datasets presented in this study can be found in online repositories. The names of the repository/repositories and accession number(s) can be found at: https://www.ncbi.nlm.nih.gov/bioproject/PRJNA934347.

## Ethics statement

The study involving human participants were reviewed and approved by University of Florida IRB1. The patients/participants provided their written informed consent to participate in this study.

## Author contributions

AF, KA, and YW performed the experiments. AF, MS, and WD contributed to the conception and design of the study. AF, JA, and WD analyzed the data. AF, JS, JA, and WD wrote the original draft. AF, JS, JA, KA, YW, MS, and WD reviewed and edited the manuscript. All authors contributed to the article and approved the submitted version.

## Funding

The Saskatchewan Pulse Growers, SK, Canada, funded this study. AF was a recipient of a Saudi Arabian Cultural Mission graduate scholarship. This work was supported by the USDA National Institute of Food and Agriculture, Hatch 1022000. The authors declare that this study received funding as in-kind microbiota analysis from Lallemand Health Solutions Inc. The funder was not involved in the study design and data collection of this article, or the decision to submit it for publication.

## Conflict of interest

Author JA, employed with Lallemand Health Solutions Inc., assisted with the analysis and interpretation of the microbiota data and in writing the microbiota methods and results.

The remaining authors declare that the research was conducted in the absence of any commercial or financial relationships that could be construed as a potential conflict of interest.

## Publisher’s note

All claims expressed in this article are solely those of the authors and do not necessarily represent those of their affiliated organizations, or those of the publisher, the editors and the reviewers. Any product that may be evaluated in this article, or claim that may be made by its manufacturer, is not guaranteed or endorsed by the publisher.

## Supplementary material

Supplementary Figure 1 Serum levels of *p*-cresyl sulfate (PCS), indoxyl sulfate (IS), phenylacetylglutamine (PAG), and trimethylamine *N*-oxide (TMAO) pre- and post-pea hull fiber. No statistically significant differences were observed.

SUPPLEMENTARY FIGURE 2 Taxonomic classification by genus level by study timepoint: baseline (1K), intervention 1(2K), washout (3K), intervention 2 (4K).

SUPPLEMENTARY FIGURE 3 Evenness (Pielou) and alpha-diversity (Faith) metrics between treatments; Pea hull fiber (Fiber) vs. Control. No statistically significant difference was observed.

SUPPLEMENTARY FIGURE S4 Beta diversity by Weighted **(A)** and Unweighted **(B)** UniFrac Principal Coordinates Analysis (PCoA) of the fecal microbiome data sets of participants receiving hemodialysis received fiber snacks and control snacks. Large overlap represents similar beta diversity between **A**) control (red) and **B**) pea hull fiber (blue).

SUPPLEMENTARY FIGURE S5 PiratePlots of all taxa distinguishing the consumption of pea hull fiber (Fiber) from usual diet during baseline and washout. The taxa are listed as follows: **(A)** *Coprococcus*, **(B)** *Lactobacillus*, **(C)** *Ruminococcus*, **(D)** *Gemmiger*, **(E)** *Collinsella*, **(F)** Mogibacteriaceae.
