# Supplementary figures and images for: Pea hull fiber supplementation does not modulate uremic metabolites in adults receiving hemodialysis: a randomized, double-blind, controlled trial
Source: Front Nutr. 2023 Jun 30;10:1179295. doi: 10.3389/fnut.2023.1179295 (PMC10349378; doi:10.3389/fnut.2023.1179295)

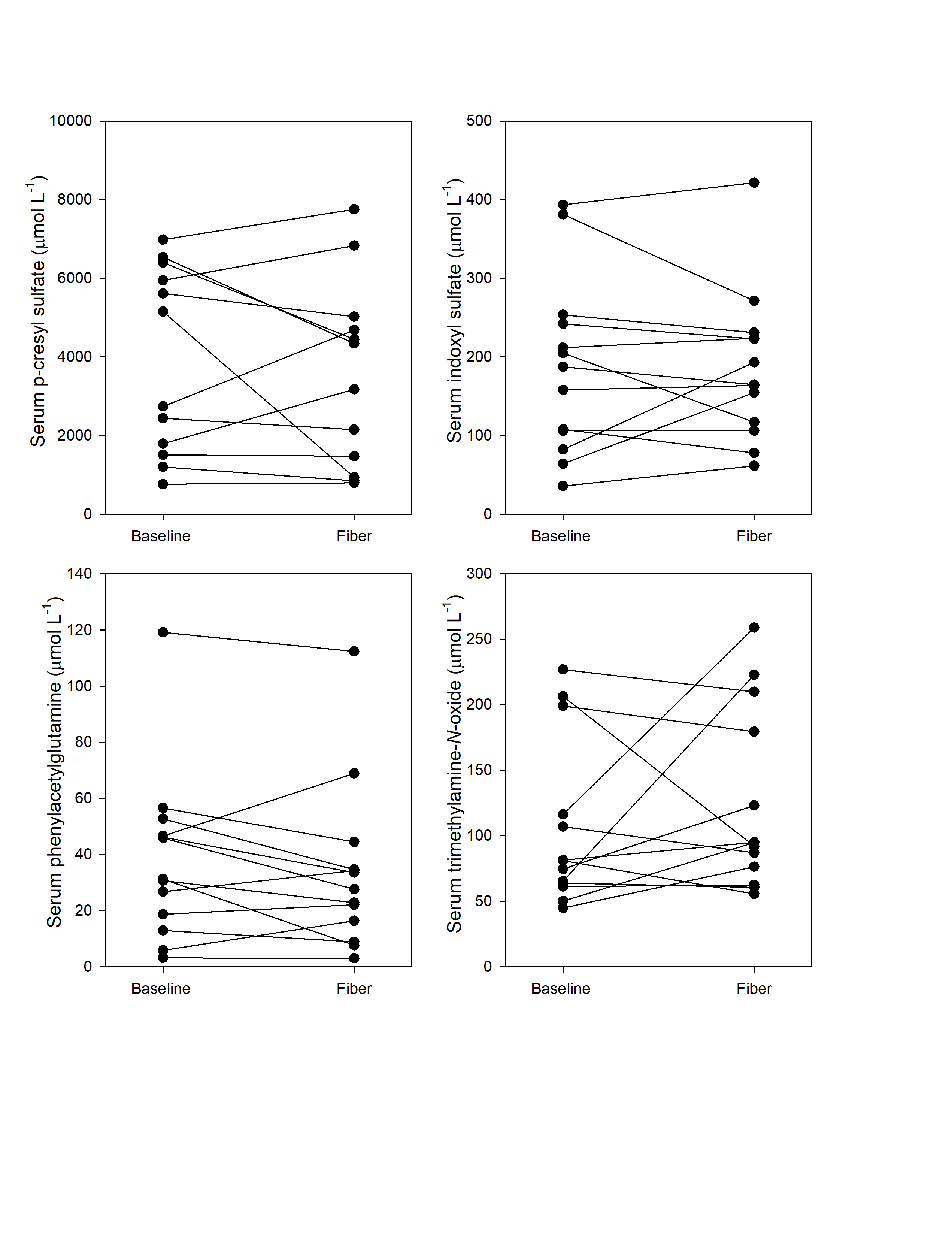

Supplement: Supplementary Figure 1 — Serum levels of p-cresyl sulfate (PCS), indoxyl sulfate (IS), phenylacetylglutamine (PAG), and trimethylamine N-oxide (TMAO) pre- and post-pea hull fiber. No statistically significant differences were observed. [file Image_1.jpeg]

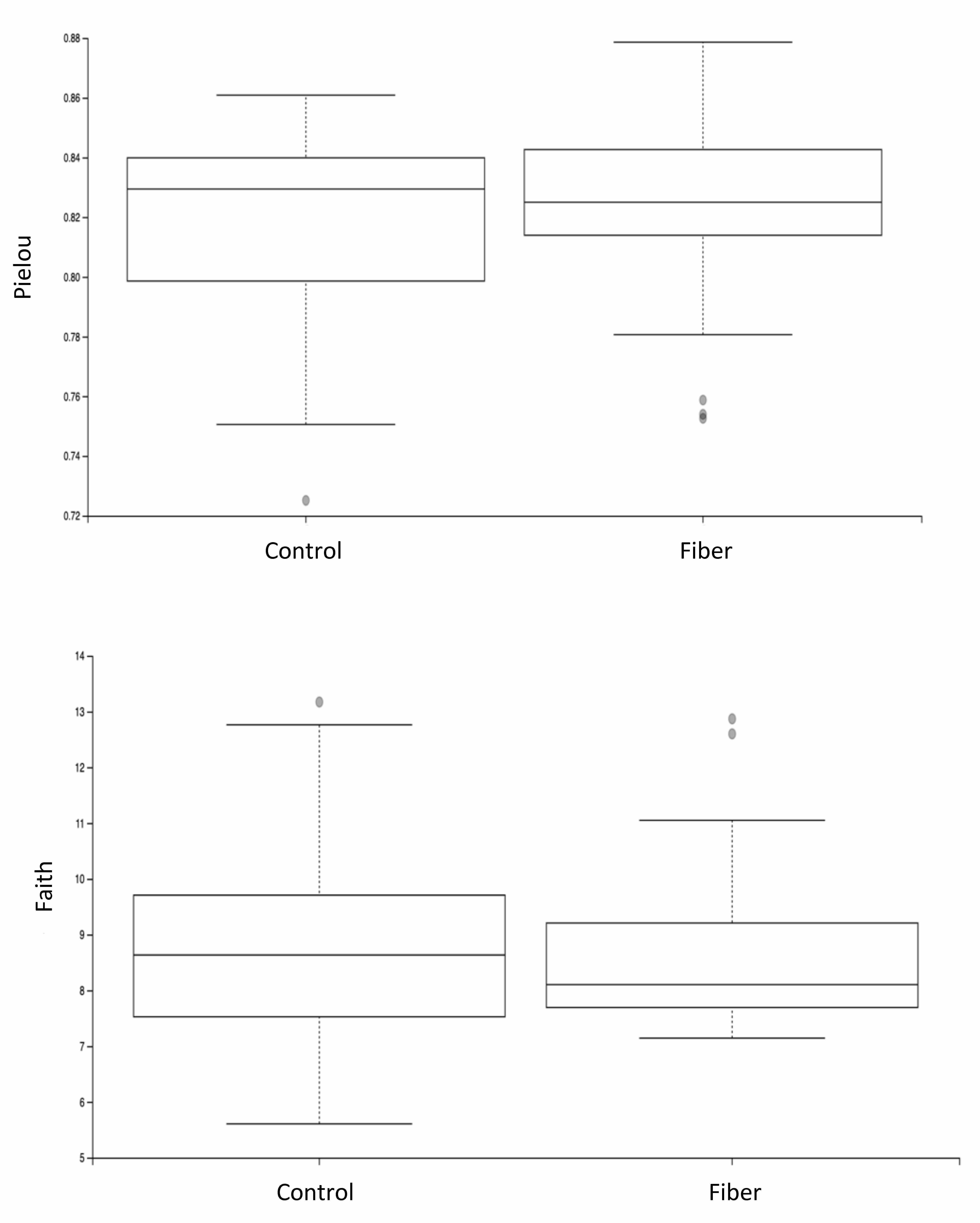

Supplement: SUPPLEMENTARY FIGURE 3 — Evenness (Pielou) and alpha-diversity (Faith) metrics between treatments; Pea hull fiber (Fiber) vs. Control. No statistically significant difference was observed. [file Image_3.jpeg]

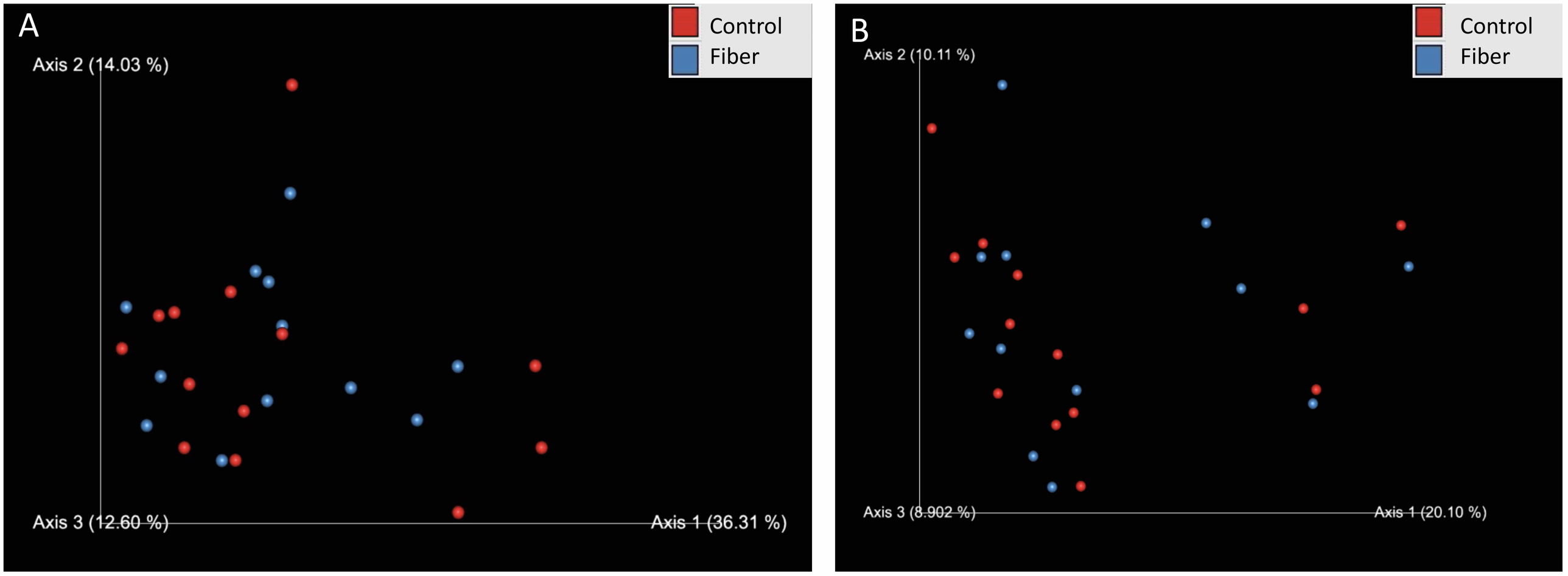

Supplement: SUPPLEMENTARY FIGURE S4 — Beta diversity by Weighted (A) and Unweighted (B) UniFrac Principal Coordinates Analysis (PCoA) of the fecal microbiome data sets of participants receiving hemodialysis received fiber snacks and control snacks. Large overlap represents similar beta diversity between A) control (red) and B) pea hull fiber (blue). [file Image_4.jpeg]

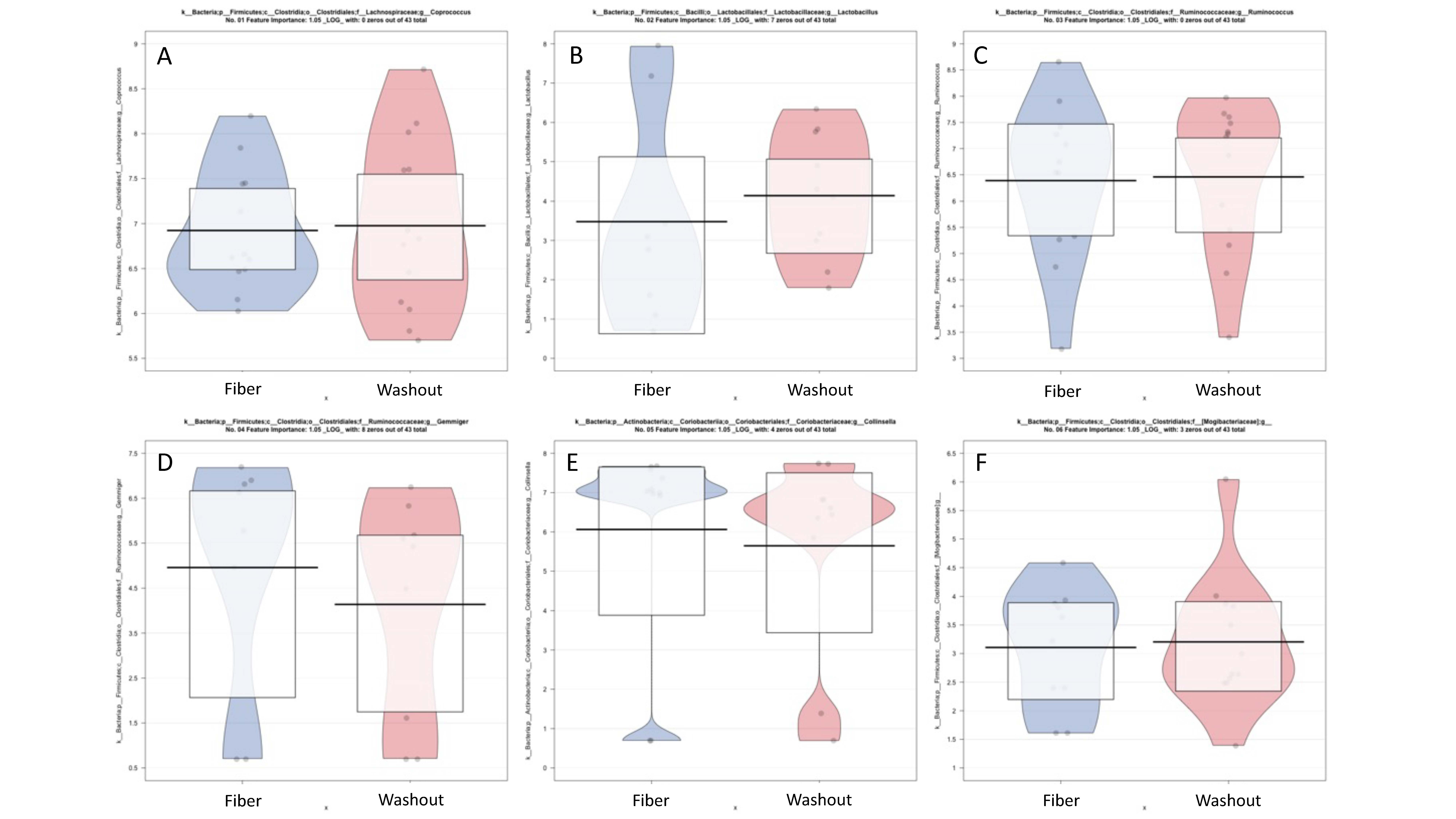

Supplement: SUPPLEMENTARY FIGURE S5 — PiratePlots of all taxa distinguishing the consumption of pea hull fiber (Fiber) from usual diet during baseline and washout. The taxa are listed as follows: (A) Coprococcus, (B) Lactobacillus, (C) Ruminococcus, (D) Gemmiger, (E) Collinsella, (F) Mogibacteriaceae. [file Image_5.jpeg]
