# Supplementary material for: Pea hull fiber supplementation does not modulate uremic metabolites in adults receiving hemodialysis: a randomized, double-blind, controlled trial
Source: Front Nutr. 2023 Jun 30;10:1179295. doi: 10.3389/fnut.2023.1179295 (PMC10349378; doi:10.3389/fnut.2023.1179295)
Supplement: Supplementary file 6 [file Table_1.pdf]

Supplementary Table 1. Background energy and nutrient intake of participants receiving hemodialysis.

	Baseline	Fiber	Control	Washout
Energy (kcal)	1786 ± 143	1699 ± 211	1931 ± 204	1956 ± 309
Carbohydrate (g)	224 ± 19	200 ±27	200 ± 21	225 ± 38
Protein (g)	68 ± 7	64 ± 6	82 ± 11	75 ± 10
Fat (g)	67 ± 7	67 ± 11	84 ± 11	82 ± 15
Dietary Fiber (g)	11 ± 1	11 ± 2	10 ± 1	12 ± 2
Iron (mg)	13 ± 3	12 ± 2	12 ± 2	12 ± 2
Magnesium (mg)	119 ± 15	117 ± 12	122 ± 19	121 ± 13
Phosphorous (mg)	583 ± 52	621 ± 72	655 ± 112	681 ± 83
Potassium (mg)	1246 ± 131	1159 ± 154	1288 ± 215	1384 ± 167
Sodium (mg)	2932 ± 315	2869 ± 486	3315 ± 491	3104 ± 423

Data presented as mean ± SD. No significant differences.
